# Broken Promises? On the Continued Challenges Faced in Catalytic Hydrophosphination

**DOI:** 10.1021/acscatal.2c03144

**Published:** 2022-08-22

**Authors:** Samantha Lau, Thomas M. Hood, Ruth L. Webster

**Affiliations:** Department of Chemistry, University of Bath, Bath BA2 7AY, U.K.

**Keywords:** hydrophosphination, hydropnictogenation, phosphines, catalysis, P−C bond formation

## Abstract

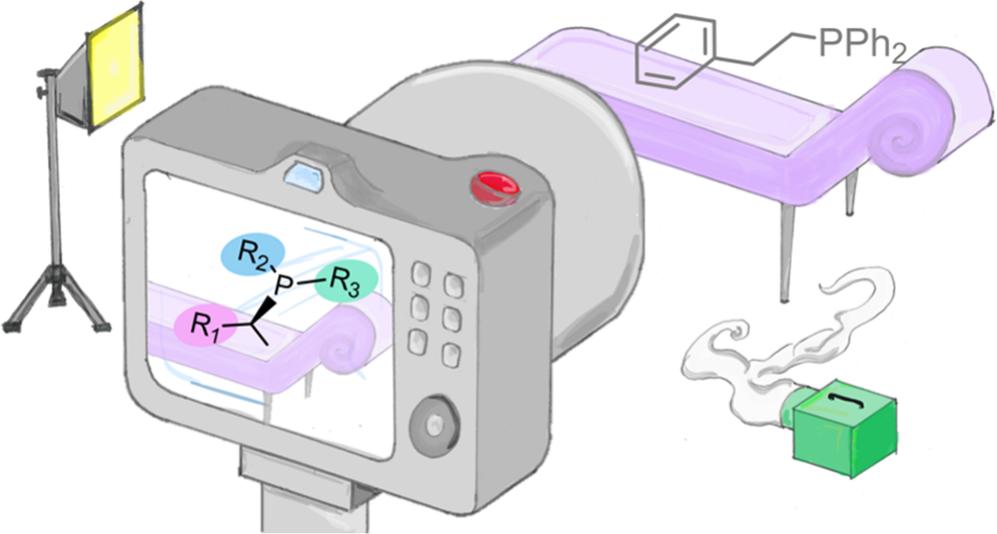

In this Perspective, we discuss what we perceive to be the continued challenges faced in catalytic hydrophosphination chemistry. Currently the literature is dominated by catalysts, many of which are highly effective, that generate the same phosphorus architectures, e.g., anti-Markovnikov products from the reaction of activated alkenes and alkynes with diarylphosphines. We highlight the state of the art in stereoselective hydrophosphination and the scope and limitations of chemoselective hydrophosphination with primary phosphines and PH_3_. We also highlight the progress in the chemistry of the heavier homologues. In general, we have tried to emphasize what is missing from our hydrophosphination armament, with the aim of guiding future research targets.

## 1 Introduction

**Figure 1:**
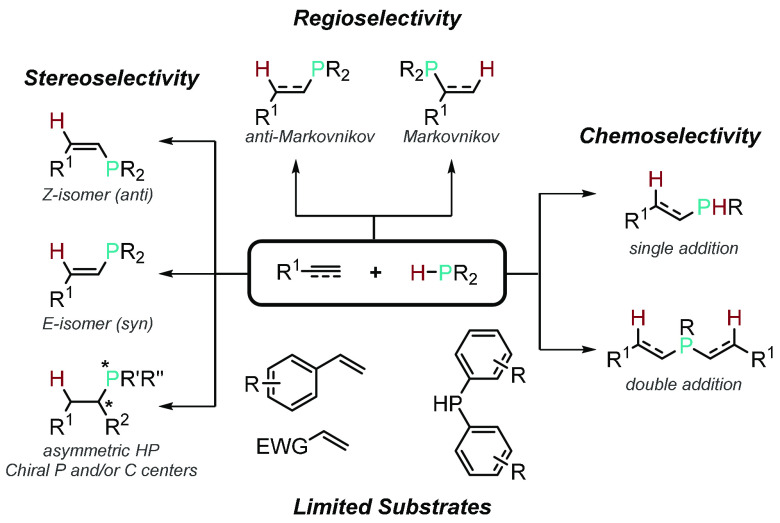
Current challenges in catalytic hydrophosphination reactions.

Catalytic hydrophosphination (HP) reactions provide an attractive route to form new P–C bonds under atom-economical conditions. This supposed synthetic simplicity has galvanized research in this area to meet the demand for phosphorus-containing ligands and substrates in the fine-chemical, pharmaceutical, and agricultural industries.^1−5^ Indeed, some notable (and otherwise challenging) hydrophosphination protocols have been reported in the patent literature,^6^ including the HP of unactivated substrates such as cyclooctadiene (COD) and limonene to generate 9-phosphabicyclo[3.3.1]nonane and 4,8-dimethyl-2-phosphabicyclo[3.3.1]nonane, respectively. The HP of these unactivated substrates requires handling of PH_3_ under pressure at high temperature and is often radical-mediated. However, in general a number of challenges are still pervasive in catalytic HP reactions: (*i*) regioselectivity in the form of Markovnikov and anti-Markovnikov products, (*ii*) stereoselectivity, (*iii*) chemoselectivity when using primary phosphines, and (*iv*) reactivity involving unactivated substrates and phosphines as the reagents (Figure 1).

Compared to catalytic HP, catalytic hydroamination is a more established field. Great strides have already been taken to overcome similar selectivity issues and, importantly, to produce synthetically relevant molecules.^7−9^ Although there are still obstacles within hydroamination chemistry, notable recent examples include work by Knowles and co-workers on intermolecular hydroamination of unactivated alkenes with cyclic and acyclic secondary amines to give exclusively anti-Markovnikov products under photocatalytic conditions.^10^ The seminal work by Bertrand and co-workers in 2008 synthesized imines, enamines, and allyl amines from the hydroamination of alkynes or allenes with the simplest of amines, ammonia,^11^ which in itself is still a very challenging avenue of hydroamination research.^12^

HP reactions have, thus far, not emulated similar indirect hydroamination reactions, for example, work from Baran and co-workers disclosing the hydroamination of alkenes starting from nitro(hetero)arenes^13^ or work by Buchwald and co-workers on asymmetric hydroamination of unactivated internal alkenes starting from hydroxylamine esters.^14^ In part this is due to the lack of access to analogous starting phosphine feedstocks. Simply going down the pnictogen group from nitrogen to phosphorus has proven to be nontrivial for analogous hydrofunctionalization reactions, as the chemist simply does not have access to the same chemical toolbox of reactions that hydroamination proffers.

This Perspective aims to review state of the art catalytic HP reactions that in part address the aforementioned challenges and is not a comprehensive history of HP^15−22^ or indeed the adjacent phosphination,^23^ hydrophosphinylation, and hydrophosphonylation^24−27^ reactions, for which there are numerous excellent reviews already. We summarize the current limitations of HP reactions, and in addition, we hope to draw parallels and highlight any lessons that can be applied from HP reactions toward the heavier hydropnictogenation reactions. However, after decades of active research into HP reactions, including work from our group,^28−35^ the progress in improving even regioselectivity issues has been incremental. We now must ask: is HP the most viable, atom-economical, synthetic route to obtain novel phosphorus precursors?

## 2 Hydrophosphination

### 2.1 Regioselectivity

#### 2.1.1 Intermolecular HP

**Scheme 1:**
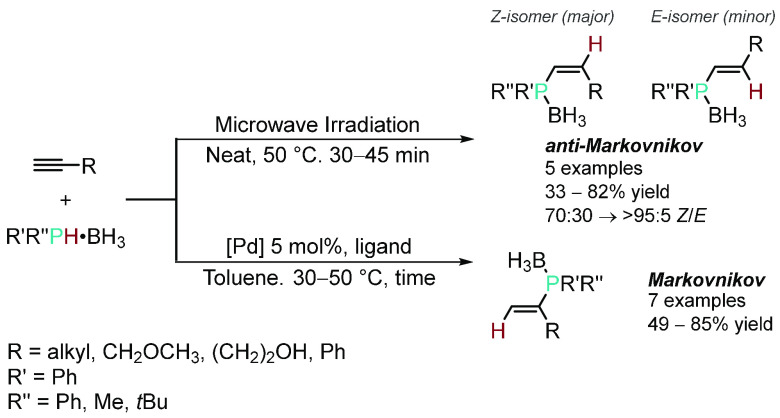
Divergent Regioselective HP of Terminal Alkynes with Phosphine-Boranes under Palladium Catalysis or Microwave Irradiation

To date, there is a paucity of literature on the selective formation of the more synthetically challenging Markovnikov product from catalytic HP. Instead, the anti-Markovnikov product is almost exclusively formed irrespective of the catalytic system, i.e., different metals, different ligand scaffolds, different solvents and temperatures. In 2003, Beletskaya and co-workers reported the HP of six alkenylalkyl ether substrates with diphenylphosphine mediated by either a nickel or palladium precatalyst at 80 °C to give selectively the Markovnikov product in good yields.^36^ Here an activated alkene was still required, and the phosphine was limited to Ph_2_PH. In the same year, Mimeau and Gaumont demonstrated the switchable addition of the P–H bond of protected secondary phosphines across terminal alkynes to give either the *Z*-selective anti-Markovnikov product under microwave irradiation or the Markovnikov product when the HP was mediated by a Pd catalyst (Scheme 1).^37^ During the screening process, when diphenylphosphine-borane was reacted with 1-octyne, various Pd precatalysts were used, and all showed selective formation of the Markovnikov product, suggesting that the regioselectivity is independent of the ligand scaffold around the Pd center—although the overall yield varied from moderate to excellent. However, changing the secondary phosphine-borane to methyl(phenyl)phosphine-borane resulted in a decrease in the regioselectivity. Unfortunately, the scope of this investigation was limited, so the origin of the regioselectivity could not be elucidated further by the authors. Following up this work, Gaumont and co-workers reported the same regioselectivity for the HP of 1-ethynylcyclohexene but also installing a stereogenic P center when the reaction was mediated using a chiral Pd catalyst, with the best result giving 70% conversion and 42% *e.e.*, representing the first example of an HP reaction forming a vinylphosphine with a stereogenic P center.^38^

**Scheme 2:**
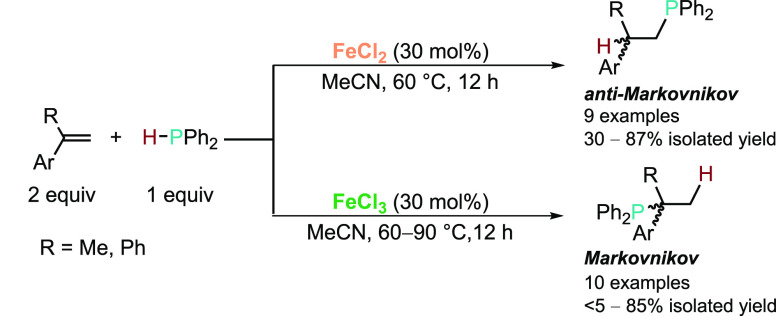
Divergent Regioselective HP of Styrene Derivatives with Ph_2_PH Mediated by FeCl_2_ and FeCl_3_

Moving away from the Noble metals, in 2013 Gaumont and co-workers were able to further demonstrate switchable regioselectivity for the HP of a number of alkenyl arenes using inexpensive and benign iron salts (Scheme 2).^39^ With FeCl_2_ salt (30 mol %) in acetonitrile at 60 °C, the anti-Markovnikov product was always selectively formed in excellent yield for terminal alkenyl arenes. However, for 1,1-disubstituted alkenyl arenes, poor yields were obtained even upon heating to 90 °C. Impressively, simply changing the precatalyst to FeCl_3_ salt (30 mol %) resulted in the formation of the complementary Markovnikov products. Furthermore, increasing the temperature to 90 °C allowed access to the desired Markovnikov products for the HP of 1,1-disubstituted alkenyl arenes in good to excellent yields. To date, this study still represents one of the most elegant and simple catalytic systems to access the Markovnikov products from the HP of styrene derivatives.

**Scheme 3:**
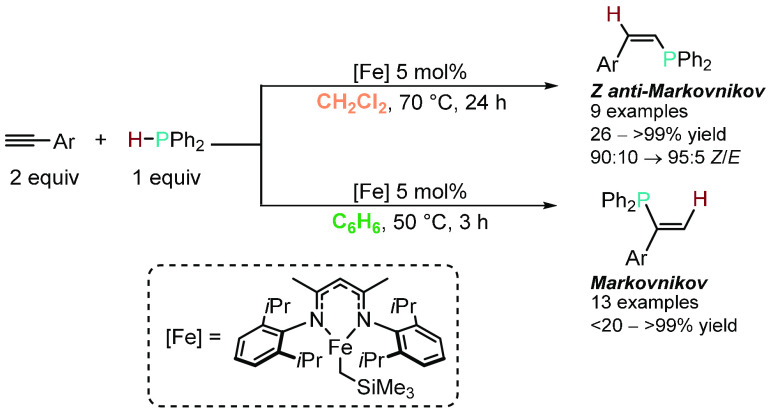
Divergent Regioselective HP of Terminal Alkynes with Ph_2_PH in Different Solvents

In 2017, we reported the HP of terminal alkynes with an iron(II) β-diketiminate precatalyst (Scheme 3).^32^ When HP was performed in *dichloromethane* at 70 °C the *Z*-selective anti-Markovnikov product was formed. However, when HP was performed in *benzene* at 50 °C, the Markovnikov product was formed. Both transformations used the same Fe(II) precatalyst. Preliminary mechanistic studies indicated that the different oxidation states of iron were the origin of the divergent selectivity, with the Markovnikov addition showing involvement of radicals and retention of the Fe(II) oxidation state.

#### 2.1.2 Intramolecular HP

**Scheme 4:**
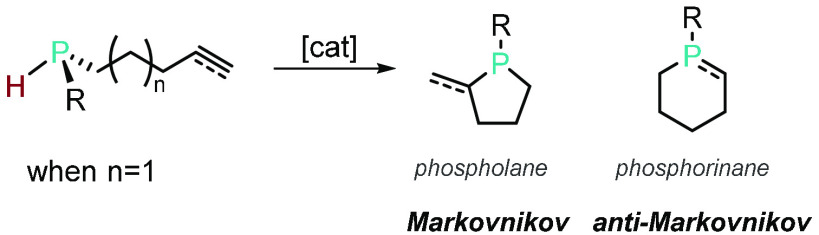
Intramolecular HP as a Strategy to Favor Markovnikov or Anti-Markovnikov Addition

Having both the unsaturated moiety and P–H bond on the same molecule can help bias the system to undergo anti-Markovnikov or Markovnikov addition because certain ring sizes are favored (Scheme 4). Early examples by Marks and co-workers disclosed the intramolecular HP of primary and secondary phosphinoalkynes and -alkenes mediated by lanthanide complexes.^40−42^ Markovnikov addition was favored to give the five-membered phospholane as the major product, with the anti-Markovnikov six-membered phosphorinane product detected as the minor product that forms under noncatalytic conditions. Furthermore, in 2016 we reported the first Fe(II)-mediated HP of nonactivated primary phosphinoalkenes and -alkynes to selectively give the Markovnikov addition products.^29^

**Scheme 5:**
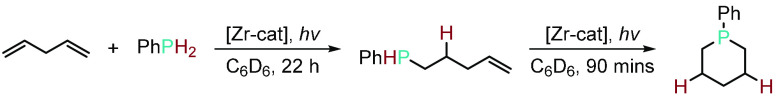
Sequential HP of 1,4-Pentadiene

To date, there are only a handful of reports on intramolecular HP simply because synthesizing these phosphinoalkyne and -alkene starting materials is nontrivial. However, in 2018 Waterman and co-workers demonstrated the sequential intermolecular HP of 1,4-pentadiene with PhPH_2_ to give the corresponding anti-Markovnikov alkenylphosphine.^43^ The alkenylphosphine then underwent intramolecular anti-Markovnikov HP to give the six-membered phosphorinane product (Scheme 5). This sequential inter- then intramolecular HP is an attractive route to form these P-heterocycles but is currently substrate-limited.

### 2.2 Stereoselectivity

**Scheme 6:**
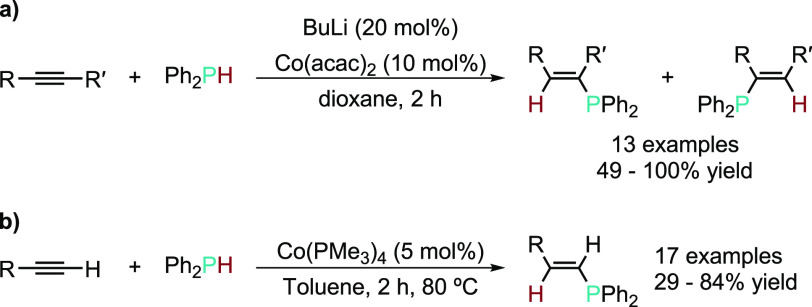
Cobalt-Catalyzed HP of Alkynes

Product distributions due to stereoselective catalytic HP can fall into two categories; *Z* or *E* isomers from anti-Markovnikov addition of the P–H bond across alkynes or from asymmetric HP to generate products containing chiral phosphorus and/or carbon centers. As mentioned, most HP reactions give the anti-Markovnikov product, and generally for the HP of alkynes, the P–H bond is added in an anti fashion to furnish the *Z* isomer as the major product (vide supra).^44^ A rare example of syn addition was reported by Oshima and co-workers on the HP of internal and terminal alkynes using Ph_2_PH mediated by [Co(acac)_2_] (10 mol %)/BuLi (20 mol %) to give the *E* isomer as the major product (Scheme 6a).^45^ In 2018, Shanmugam, Shanmugam, and co-workers reported a well-defined [Co(PMe_3_)_4_] precatalyst to also effect the *E*-selective HP of internal and terminal alkynes with an emphasis on elucidating the mechanism (Scheme 6b).^46^

**Scheme 7:**
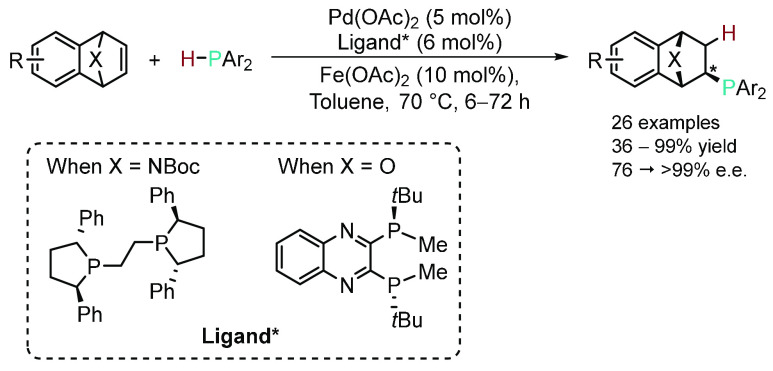
Asymmetric HP of Heterobicyclic Alkenes to Furnish Novel Chiral Phosphine Ligands

In principle, asymmetric HP is an incredibly powerful synthetic route to access the next generation of chiral phosphine ligands. This area of HP has been dominated by palladium complexes bearing chiral chelating auxiliaries.^47−66^ However, there have been early examples of nickel-catalyzed^67,68^ and organocatalytic^69−71^ asymmetric HP. More recently Wang and co-workers reported the asymmetric HP of numerous azabenzonorbornadiene and oxabenzonorbornadiene substrates with different secondary biarylphosphines mediated by palladium precatalyst and Fe(OAc)_2_ as a substoichiometric additive (Scheme 7).^72^ This research deviated from the usual α,β-unsaturated substrates (ketones and imines) used in previous asymmetric HP reactions involving palladium (vide supra) and instead involved a “non-electronically-activated double bond”. The authors stipulated that the proximity of this double bond to the high angle strain associated with the cyclic heteroatom allowed access to reactivity to form the products in moderate to excellent yields with excellent enantioselectivity. A proof of concept was further demonstrated by the group using their novel chiral phosphine ligands in asymmetric addition of phenylboronic acid to aryl aldehydes.

**Scheme 8:**
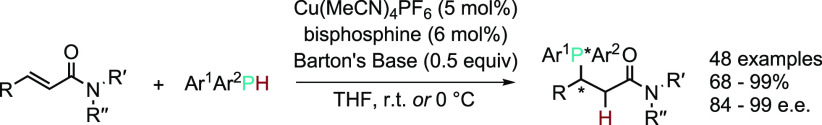
Cu-Catalyzed Asymmetric HP of α,β-Unsaturated Amides

In 2020, Yin and co-workers disclosed the Cu-catalyzed asymmetric HP of α,β-unsaturated amides with Ph_2_PH to furnish C-chiral products in good to excellent yields with high enantioselectivity (Scheme 8).^73^ More impressively, using unsymmetrical ArPhPH, six new products containing both P-chiral and C-chiral centers were formed, albeit in moderate isolated yields with moderate diastereoselectivity but high enantioselectivity.

**Scheme 9:**
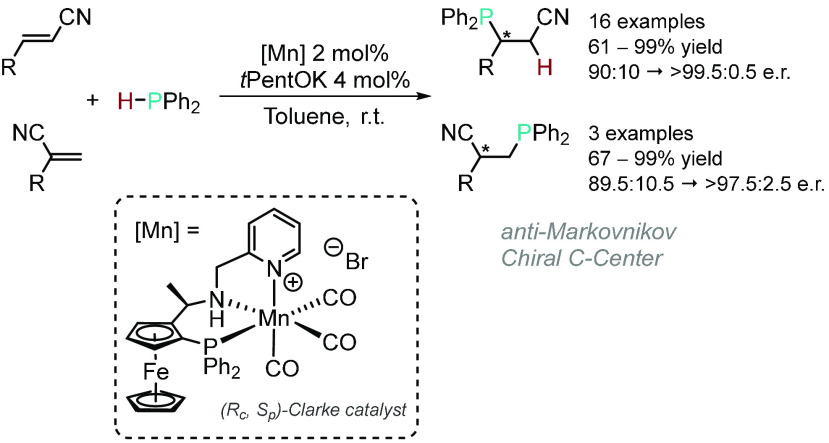
Mn(I)-Catalyzed Asymmetric HP of Vinyl Nitriles and α,β-Unsaturated Nitriles^76^

Furthering the progress originally reported by Glueck and co-workers on the HP of vinyl nitriles,^74,75^ Harutyunyan and co-workers demonstrated the Mn(I)-catalyzed asymmetric anti-Markovnikov HP of vinyl nitriles and α,β-unsaturated nitriles using Clarke’s chiral proligand (Scheme 9).^76^ Mechanistically, the authors suggested that the HP proceeded via metal–ligand cooperation, and the origin of the enantioselectivity was assessed by computational studies. The same group expanded the HP strategy toward α,β-unsaturated phosphine oxides to form a range of enantiopure 1,2-bisphosphine ligands.^77^

### 2.3 Chemoselectivity

**Scheme 10:**
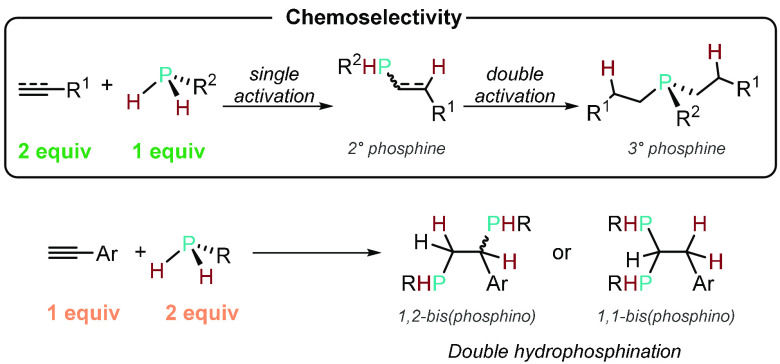
Chemoselective Single Addition or Double Addition of RPH_2_ to an Unsaturated Substrate to Furnish the 2° or 3° Phosphine, Respectively, versus Double Hydrophosphination

Using secondary phosphines (R^1^R^2^PH) for HP reactions allows the formation of tertiary-phosphine-containing products with no possibility of a second hydrophosphination event occurring. However, if primary phosphines (RPH_2_) are used, then the secondary phosphine product that is initially formed could undergo competing HP with the unsaturated substrate. This reactivity is strictly different from the reaction of 2 equiv of phosphine (R^1^R^2^PH, where R = H or alkyl/aryl) with 1 equiv of substrate bearing a triple-bond moiety. For consistency with the literature, we will follow naming the latter reaction as double hydrophosphination (itself an elusive and challenging transformation^78−81,33^) and the chemoselectivity issue arising from using a primary phosphine as single and double activation, respectively (Scheme 10).

**Scheme 11:**
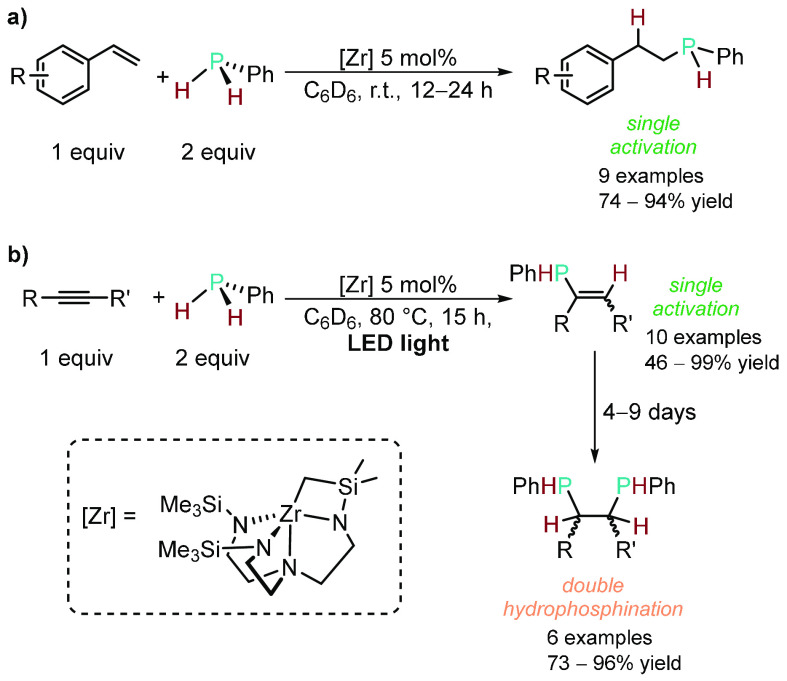
(a) Chemoselective Formation of 2° Phosphine from Single Addition of PhPH_2_ to Styrene Derivatives; (b) Sequential Single Addition of PhPH_2_ to Internal Alkynes Followed by a Second HP with PhPH_2_ to Furnish 1,2-Bis(phosphino) Substrates

There are a few examples of HP reactions involving primary phosphines, with most of the reports restricted to styrene as the substrate and PhPH_2_ as the primary phosphine.^82−85,30,86,87^ To date, one of the best examples was reported in 2014 by Waterman and co-workers on the HP of alkenes and dienes catalyzed by a triamidoamine-supported zirconium complex under mild conditions with 2:1 PhPH_2_:substrate stoichiometry to selectively form the secondary phosphine product (Scheme 11a).^88^ Progress on Zr-catalyzed single activation of RPH_2_ has been made since then by Waterman and co-workers, including the use of a chiral secondary phosphine with various alkenes^89^ and, more recently, light-driven Zr catalysis using different secondary phosphines (RPH_2_, where R = cyclohexyl, phenyl, or mesityl) with much shorter reaction times and slightly expanded scope relative to the seminal work.^43^ There are even fewer examples of HP using primary phosphines with alkynes.^90,91^ One reason for this could be competitive binding of the generated vinylphosphine product (from the first activation event) to the HP catalyst over the alkyne substrate.^92−97^ However, Bange and Waterman expanded the Zr-catalyzed HP procedure to internal alkynes with PhPH_2_.^98^ They found that the formation of the single-activation vinylphosphine product occurs first; this product can be isolated (although with poor *E*/*Z* selectivity) or proceed to react with a second equivalent of PhPH_2_ (double hydrophosphination) to obtain 1,2-bis(phosphino)alkane products after a protracted time frame (Scheme 11b). The same report also disclosed the reaction of PhPH_2_ with acetylene to furnish 1,2-bis(phenylphosphino)ethane in 65% yield, which is impressive because the acetylene was postulated to deactivate the catalyst and the zirconium catalyst was known to have poor reactivity with terminal alkynes.^99,100^

### 2.4 Heteroallenes and PH_3_

**Scheme 12:**
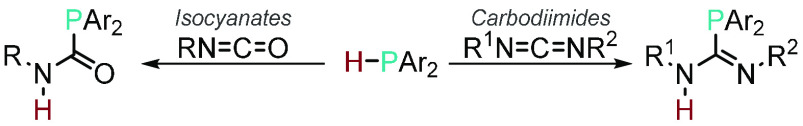
Single HP of Isocyanates and Carbodiimides

Heteroallenes as substrates have been far less explored in HP reactions due to the propensity for unwanted side reactivity such as cyclotrimerization^101^ and selectivity issues with either single or double insertion. The products offer novel ligand scaffolds that could be employed in catalysis.^102,103^ Examples include HP of carbodiimides (RN=C=NR′) and isocyanates (RN=C=O), and these transformations have been dominated by main group-,^104,105^ lanthanide-,^106−109^ and actinide-based^110,111^ catalysis, with some p-block catalysis^112−114^ being reported in the past decade (Scheme 12).

In 2017, Kays and co-workers reported the first transition-metal-catalyzed HP of isocyanate using Fe(II) precatalysts.^115^ The expected single insertion of RN=C=O into Ph_2_PH was observed as one of the products, but an unprecedented second product was also identified as the double insertion of RN=C=O into Ph_2_PH to form a new family of phosphinodicarboxamide products. By tailoring the steric bulk of the R groups of the isocyanates, the single-insertion product can be exclusively formed with more bulky substituents. Changing the solvent from C_6_D_6_ to THF also renders exclusively the single-insertion product. Since 2017, expansion into other transition metal complexes to achieve the successful HP of heteroallenes and heterocumulenes has been reported.^116−118^

More recently, Nakazawa and co-workers reported the HP of a variety of RN=C=O (R = aryl or alkyl) with Ph_2_PH without any solvent at room temperature and short reaction times (0.25–12 h) to afford the single-insertion products.^119^ In addition, the single HP of phenylisocyanate with the primary phosphine PhPH_2_ was also achieved to give the product in 71% yield, although this required 3 days for completion.

**Scheme 13:**
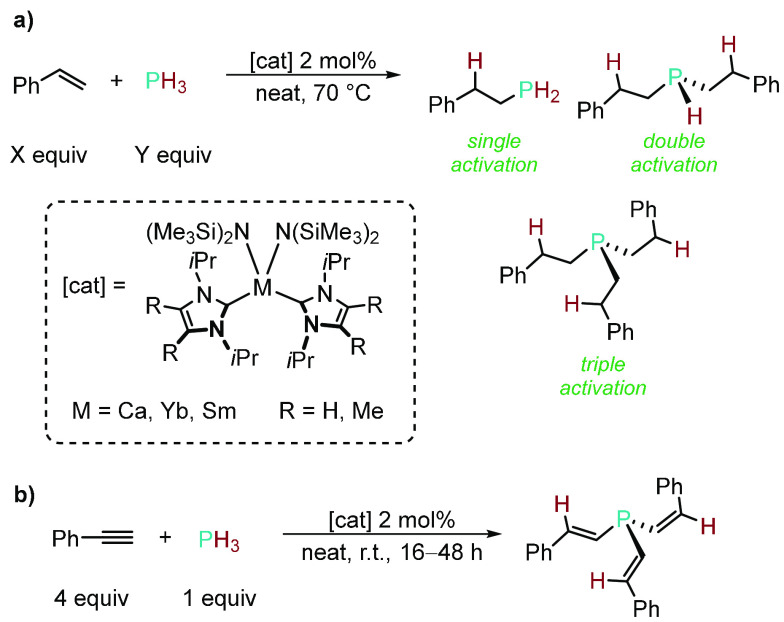
(a) Regio- and Chemoselective HP of Styrene with PH_3_ by Altering the Substrate:PH_3_ Ratio; (b) Exclusive Formation of Tris(*Z*-styryl)phosphine from HP of Phenylacetylene with PH_3_

Catalytic HP reactions using PH_3_ are niche. The high toxicity and specialized equipment required to use this gas have resulted in very few examples being reported to date.^120^ In the 1990s, Pringle and co-workers led the way using platinum complexes to effect the HP of formaldehyde,^121,122^ ethyl acrylate,^123^ and acrylonitrile^124,125^ with PH_3_—for each substrate, the tertiary phosphine products can be selectively formed over time. Interestingly, the secondary phosphine products were observed by ^31^P NMR spectroscopy in these reactions, but access to these secondary phosphine products would therefore require careful monitoring of the reaction time and nontrivial separation methods. However, in 2019 Trifonov and co-workers reported the regio- and chemoselective HP of styrene with PH_3_ mediated by M(II) (M = Ca, Yb, Sm) bis(amido) complexes supported by N-heterocyclic carbene ligands.^126^ Controlling the ratio of styrene to PH_3_, the anti-Markovnikov secondary or tertiary phosphine could be formed selectively (Scheme 13a). The substrate scope was expanded to 2-vinylpyridine, unfortunately with a loss of chemoselectivity and longer reaction time. Other alkene substrates, including 1-nonene, 2,3-dimethylbutadiene, cyclohexene, and norbornene, did not react with PH_3_. Instead, the HP of phenylacetylene with PH_3_ was achieved to give exclusively tertiary tris(*Z*-styryl)phosphine regardless of the ratio of substrate to PH_3_ (Scheme 13b). Control experiments showed that the free N-heterocyclic carbenes were also capable of mediating these HP reactions with PH_3_, albeit with poorer chemoselectivity and again slower reactivity. Nevertheless, this work shows very promising results in using PH_3_ to form simple but synthetically useful secondary phosphine precursors with non-precious-metal catalysts.

In 2022, Liptrot and co-workers reported the formation of secondary and primary phosphines, including PH_3_, from reduction of the corresponding phosphorus(III) esters with pinacolborane mediated by a Cu(I) precatalyst.^127^ Subsequent HP of heterocumulenes was achieved using these phosphines generated in situ with the same precatalyst to give the single-addition products in moderate to good yields.

## 3 Limitations

### 3.1 Product Diversity

The examples outlined above are the current state of the art in HP catalysis and are absolutely worthy of note. However, it is fair to say that they are not representative of the field as a whole. In general, the products of HP share very similar structural motifs, regardless of the choice of catalyst. Indeed, most reports of HP focus on diarylphosphines and activated alkenes (likely because this is necessary to avoid any issues of regio- or chemoselectivity). For example, even a brief literature search revealed >40 reports on the anti-Markovnikov HP of styrene with Ph_2_PH. An exciting development that moves away from Ph_2_PH is Benhida and Cummins’ use of bis(trichlorosilyl)phosphine, (Cl_3_Si)_2_PH, which can be used for UV-light-mediated anti-Markovnikov HP of unactivated alkenes.^128^ The activity of this system serves to further highlight the opportunities that still exist in HP if we move away from traditional catalyst systems or if we develop more active catalysts that can compete with a UV-light-mediated reaction. The issue of anti-Markovnikov selectivity is compounded further by the fact that the thermally induced, catalyst-free HP occurs with near-perfect anti-Markovnikov selectivity.^129^ Thus, many reported HP catalysts are simply lowering the barrier toward product formation rather than allowing access to difficult-to-prepare P-containing products. One could even go as far as to say that the anti-Markovnikov-selective catalysis of diarylphosphines with activated alkenes undermines the often-cited selling point of HP. Phosphorus-containing compounds are ubiquitous in chemistry, but how many times has (2-phenylethyl)diphenylphosphine been used as a ligand, for example? The answer is only a handful, and therefore, does the demand for such products reflect the abundance of HP catalysis reported?^130−134^

Of course, the field has been expanded to included alkynes and other hetero-unsaturated species, which allows for some increased diversity in product formation, and indeed some of the products are unique and warrant additional focus (vide supra).^115,127^ For example, the vinylphosphines generated from HP of alkynes can potentially be further functionalized by HP, but the products of this transformation are ultimately usually limited to functionalized 1,2-bis(diphenylphosphino)ethane (dppe) derivatives.^81,135,136^

To build truly diverse HP products and construct molecular complexity from simple molecules, we need more potent catalysts, including catalysts that can facilitate HP of even the most unactivated substrates. The grand goal should be to use HP to expand the phosphorus pool and to access bespoke phosphines. Functionalizing a primary phosphine to generate new P*RR′R″ species would be a very powerful use of HP. However, with primary phosphines overfunctionalization becomes a problem, which then brings us to our next major stumbling block: selectivity.

### 3.2 Selectivity

The first and most obvious selectivity hurdle to overcome is the considerable anti-Markovnikov regioselectivity associated with HP. If any progress is to be made, we need catalysts that subvert this selectivity bias and reliably afford the Markovnikov products. Some Markovnikov-selective regimes have been reported (vide supra), but most still require activated substrates. One of the few exceptions to this comes from Marks and our own report on the HP of nonactivated primary phosphinoalkenes and alkynes, though we openly note that the intramolecular nature of the reaction necessitates the HP of the otherwise unactivated alkene.^41,29^ Furthermore, the elevated temperature associated with our HP catalysis means that stereoselectivity could not be achieved.

As mentioned above, controlled, chemoselective HP catalysis utilizing primary phosphines (or even PH_3_) is highly desirable. However, because of the propensity for overfunctionalization, this remains a considerable challenge. Very few catalysts are effective at chemoselectively affording the single-activation product; when they are effective, they typically (of course) form the anti-Markovnikov product. This then only leaves the issue of stereoselectivity—perhaps the most challenging yet desirable of all. Developing chiral variants of known HP catalysts is challenging enough, but the difficulty is often compounded by the need for elevated temperatures as well as evidence for radical mechanisms in HP.^32^ Notably, however, the most successful catalysts in enantioselective HP are those that can operate at low temperatures, conferring a need for more active catalysts that can operate as such. Additionally, asymmetric HP suffers from the same overarching problem discussed throughout: the need for activated substrates. The azabenzonorbornadiene and oxabenzonorbornadiene derivatives described above as asymmetric HP substrates successfully deviate from the classical unsaturated substrates (Scheme 7).^72^ However, the procedure is restricted to the familiar diarylphosphines and (while the double bond is not nonelectronically activated) utilizes strained double bonds.

**Scheme 14:**
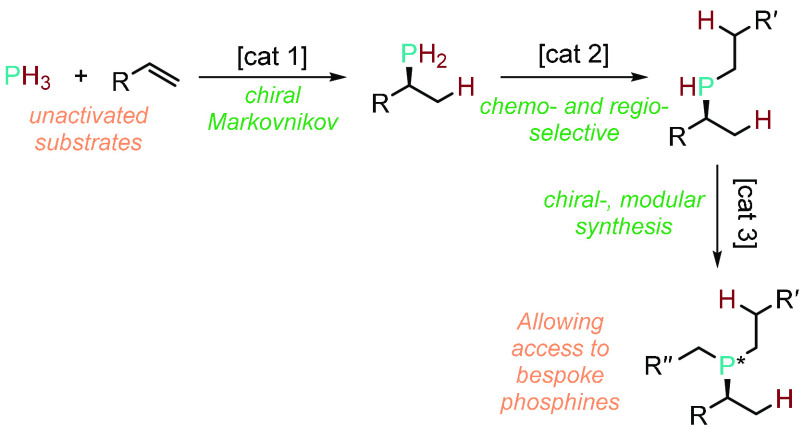
Hypothetical, Modular, and Effective Use of HP to Synthesize Highly Functionalized Bespoke Phosphines

All of these issues must be overcome individually first if there is any hope to make HP a truly useful synthetic tool. The grand goal must be generalizable HP of unactivated substrates with complete tunability of the regio-, chemo- and enantioselectivity (Scheme 14). It is an ambitious goal, but a necessary one if HP is going to be the future of P–C bond-forming chemistry. It is clear that a major breakthrough is necessary in order to use HP to access P-containing species of significant value.

## 4 Heavier-Congener Hydropnictogenation

The significant advances in main-group chemistry over the last few decades have prompted interest in the heavier pnictogen congeners and their corresponding hydropnictogenation reactions.^137−140^ Compared to the abundance of HP reports, these heavier hydrofunctionalization endeavors are still in their infancy, and the very act of catalytic hydrobismuthation (unreported to our knowledge) would be a remarkable achievement. However, while we can make a direct link between HP and the potential for novel ligand design, a relationship to applications of the heavier homologues is less clear. Furthermore, the same limitations are already emerging, and we hope that this Perspective will assist those undertaking hydropnictogenation research into striving for more diverse reactivity than is currently observed in the field of HP.

**Scheme 15:**
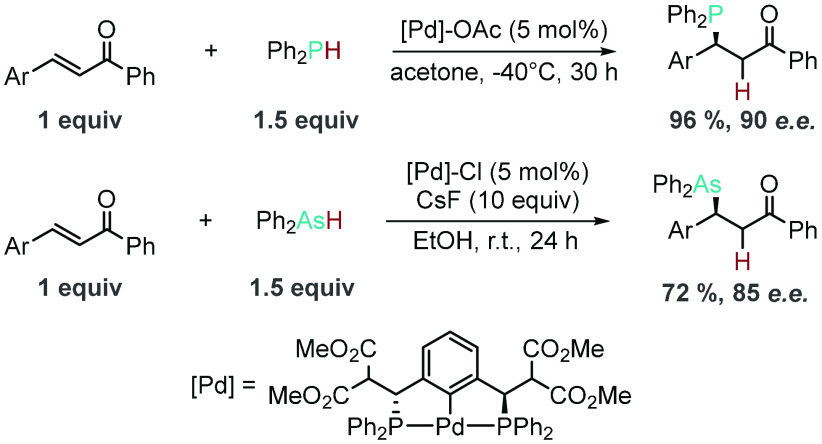
Analogous Pd-Catalyzed Hydrophosphination and Hydroarsination

Leung and co-workers have already shown that there is transferable knowledge between HP and the heavier analogues.^141^ They were able to use a very similar chiral palladium catalyst to effect the HP and hydroarsination of internal alkenes with only minor catalyst alterations: changing the ancillary ligand from acetate to chloride (Scheme 15). Both transformations were optimized to give excellent yields and high enantioselectivity, albeit with the need for an activated alkene to partner with either Ph_2_PH or Ph_2_AsH.

Waterman and co-workers have also achieved the single hydroarsination of phenylacetylene with Ph_2_AsH utilizing their triamidoamine-supported Zr catalyst (vide supra).^142^ Importantly, they also noted that the reaction occurs without the need for the zirconium catalyst when conducted in ambient lighting but required the catalyst in order to proceed in the dark. In this case the reaction afforded a 6.6:1 mixture of *cis*- and *trans*-vinylarsine products respectively. While this study was mainly a proof of concept of a then-rare hydroarsination, the selectivity observed is all too familiar with regard to HP.

**Scheme 16:**
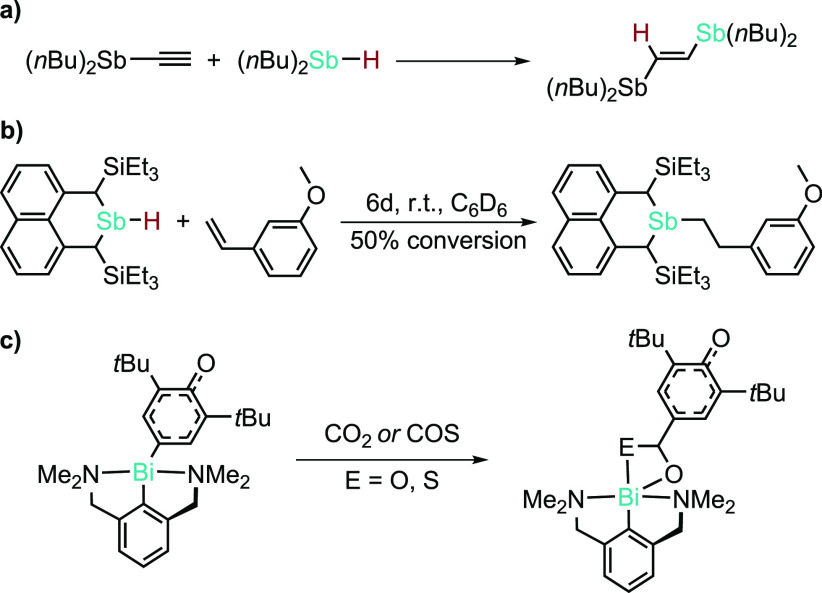
Catalyst-Free Hydrostibinations and an Arylbismuthation

Generally, reports of hydrostibination are fewer still, despite the fact that the first example was reported in 1965 (Scheme 16a).^143^ Since then, a handful of Lewis acid- or radical-initiated examples have been reported. More recently, Chitnis and co-workers reported a catalyst- and initiator-free hydrostibination by tuning the stibene backbone to stabilize the LUMO of the stibene (Scheme 16b). They later comprehensively studied the mechanism of this hydrostibination and suggested that a radical mechanism is at play.^144^ Finally, in the context of HP, it is important to note that all of the heavier-congener hydropnictogenations reported display near-perfect anti-Markovnikov selectivity.

While there have been no reports of hydrobismuthation (likely because of the difficulty in isolating bismuth hydrides), there are reports of arylbismuthation, which we would be remiss not to highlight (Scheme 16c).^145^

## 5 Conclusions

While we understand that research progress takes time, the HP community is still plagued by the same challenges addressed in Waterman’s 2016 review.^20^ For HP to reach its true potential, we need a toolbox of HP catalysts that can incrementally build complexity about a P center. Ultimately, substrate scope is the most restrictive obstacle (followed closely by the regioselectivity issue), and these obstacles need to be addressed foremost before the focus is turned toward chemo- and stereoselectivity. Although HP of (for example) styrene with diphenylphosphine can be a useful test for the proficiency of a newly designed catalyst, if we cannot effectively utilize the restricted product pool of current HP or use HP to access bespoke phosphine architectures, then we must ask ourselves whether we are making enough of an advance in chemical research. A simple way to, as a minimum, offer up the potential for novel reactivity would be to include unactivated substrates in all HP studies, e.g., 1-hexene and Cy_2_PH in combination with Ph_2_PH or styrene. At the moment, the mechanism of HP is entirely limited by catalyst design, which results in oxidative addition of the HP bond across a metal center or a σ-bond-metathesis M–PR_2_ bond-forming step, both of which dictate anti-Markovnikov regiochemistry of the reaction.^17^ However, some of the most promising emerging HP catalysts (Schemes 8, 9, and 15) do not operate via these mechanisms, and while they are currently still substrate-limited, they have begun to tackle some of the most pressing limitations of HP and provide a promising direction for future research. This leaves the field in a predicament: either tangible solutions to the outstanding problems or new, innovative approaches are a necessity if we are to access novel phosphorus architectures in an atom-economical way.
